# Systems Science and Evidence-Informed Deliberation to Mitigate Dilemmas in Situations of Dual Agency at the Hospital Level: Comment on "Dual Agency in Hospitals: What Strategies Do Managers and Physicians Apply to Reconcile Dilemmas Between Clinical and Economic Considerations"

**DOI:** 10.34172/ijhpm.2022.6940

**Published:** 2022-03-02

**Authors:** Leon Bijlmakers

**Affiliations:** Department for Health Evidence (HEV), Radboud Institute for Health Sciences (RIHS), Radboud University Medical Center, Nijmegen, The Netherlands.

**Keywords:** Diverging Values, Economic Considerations, Reconciliation Strategies, Decision Criteria

## Abstract

The article by Waitzberg et al on dual agency in hospitals reports on three strategies to mitigate dilemmas arising from conflicting clinical and economic considerations. This could be further explored by using systems science methods that allow in-depth analyses of (health) system dynamics, networks, and agent-based modelling, and that take into account local context, incentives and how institutions work. Future studies may also draw on the literature of multi-criteria decision-making and evidence-informed deliberative processes (EDPs) that are increasingly being used to optimise legitimate health benefit package design. Toolkits to assist hospital professionals in improving their decision-making need to be practical, with ample attention for the process of decision-making, including transparency, use of evidence, and opportunities for health professionals (and possibly others stakeholders) to contest or formally appeal against certain decisions.

 Waitzberg et al^1^ provide empirical insights as to how hospital professionals in Germany and Israel navigate clinical and economic considerations in their decision-making and how they mitigate dilemmas when they occur. Both countries have activity-based payment mechanisms in place under mandatory, statutory health insurance systems, with similar economic incentives for hospitals to increase not only the number of cases they treat, but also the income per patient, while containing the costs per patient. Many hospital professionals are dual agents, in the sense that on the one hand they need to ensure the safety of their patients and the provision of good quality care, while on the other hand they are co-responsible for the financial sustainability of the hospitals that employ them.

 The authors’ premise is that the economic responsibilities of these hospital professionals may conflict with their clinical responsibilities, thereby presenting dilemmas that need to be acknowledged and dealt with. Based on a thematic analysis of the results of semi-structured interviews with hospital managers, chief physicians and practicing physicians, the authors of this article identify three themes, that are common to Germany and Israel, namely: (*a*) Increasing efficiency to resolve dilemmas in cases where clinical and economic considerations are aligned; (*b*) Reshaping ward management and/or activity coding in cases where clinical and economic considerations cannot be reconciled; and (*c*) Reframing decision-making to cope with unresolvable dilemmas. For the latter strategy, which involves a change of perspective, rather than changes in clinical or managerial practices, the article provides two examples. The first involves shifting the focus of decision-making from the individual patient to a whole category of patients within a particular diagnosis-related group so as to balance out a small number of financially unattractive clinical activities with a larger number of financially attractive ones. The second example involves the development of tools or tool kits that hospital professionals with dual agency can use in their daily decision-making without having to rely on personal judgement. Even though an assessment of the frequency with which the dilemmas described occur was beyond the scope of this paper, there is little doubt that hospital professionals in other hospitals, also in other countries with activity-based payment systems, will recognise them.

 In my commentary on the article I would like to concentrate on four points. Firstly, the conceptualisation of both the clinical and the economic considerations appears somewhat narrow. Under the study limitations the authors do acknowledge that there may be other considerations than clinical and economic ones – such as social needs and managerial requirements – but from a purely clinical perspective one could consider other dimensions of the treatment provided than just ‘patient safety’ or ‘quality of care’; for example appropriate use, societal side-effects, or availability of alternative forms of treatment. Especially the latter point ties in well with the joint, multidisciplinary decision-making that Waitzberg et al identified as a strong reconciling strategy. Likewise, economic considerations could include the potential long-term economic benefit for a hospital if it were to develop a particular type of expertise and distinguish itself from other hospitals, rather than the immediate financial returns from reimbursement under the existing activity-based financing mechanism which may or may not cover the entire treatment costs. Also, the societal costs, including patient and opportunity costs – as opposed to just the costs to the service provider – may be a factor that could be considered in decision-making. The interview protocols that were used (provided by Waitberg et al in their Supplementary file 1) do not seem to provide room for such wider interpretations of clinical and economic considerations. Admittedly, at the level of an individual hospital it may not be easy to consider societal side-effects, societal costs or opportunity costs, but at the national level these are relevant points.

 My second comment pertains to the method used for data collection, which involved one-time individual interviews only. The trustworthiness of the transcripts, including the common themes identified by the researchers, would have benefited from a post-hoc validation by the interviewees, also called member checking.^2,3^ Going a step further, and also in view of the complex nature of the study topic, it might have had significant added value if the respondents were given the opportunity to share their personal views and experiences with fellow professionals and to further reflect on them. Systems thinking, and in particular the notion of complex adaptive systems and associated behaviours, can help researchers – and eventually policy makers, planners and programme implementers as well – to better understand real-world phenomena, including networks, path dependence and feedback loops.^4,5^ Complex systems abound in public health. They are “made up of heterogeneous elements that interact with one another, have emergent properties that are not explained by understanding the individual elements of the system, persist over time and adapt to changing circumstances.”^6^ Systems science methods allow in-depth analyses of system dynamics, networks, and agent-based modelling, with due attention to local context, incentives and how institutions work. While fairly new and still underutilised, there are some good recent examples in this field, for example on vaccine hesitancy,^7^ and immunisation system design.^8^ In our own study in Zambia, which involved a series of participatory action research workshops and dynamic modelling, we explored policy options for embedding a surgical mentoring initiative into Zambia’s national health policy.^9^ Admittedly, systems science is quite different from the method employed in the present paper, but given the nature and importance of the topic at hand it might be of future use to shed further light on the challenges and dilemmas that health professionals with dual agency face in their decision-making.

 My third comment is that further work on reconciliation strategies, navigating diverging considerations and mitigating dilemmas may draw on the literature of multi-criteria decision-making and evidence-informed deliberative processes (EDPs) that are increasingly being used to optimise legitimate health benefit package design. Multi-criteria decision-making refers to “a collection of formal approaches which seek to take explicit account of multiple criteria in helping individuals or groups exploring decisions that matter.”^10^ It is typically used by health systems specialists, hospital managers, health technology assessment (HTA) agencies, research organizations and the insurance and pharmaceutical industries, to support health care decision-making.^11^ Decision-making by HTA agencies, in particular on whether or not the available technologies would merit inclusion into a country’s standard health benefit package is “an intrinsically complex and value-laden political process that takes place in an environment of diverging social values and interests.”^12^ The value frameworks from which HTA agencies derive the decision criteria they use are often not made explicit; or they fall short of taking stakeholder diversity and divergence in preferences into account.^12^ EDPs have been developed to address these shortcomings by offering a practical and step-wise manner for HTA agencies to bring together relevant stakeholders and have them reflect and deliberate on values, decision criteria and the available evidence on the performance of health technologies on these criteria with a view to arrive at optimal decisions or recommendations for their in- or exclusion into the standard benefit package. EDPs typically integrate four elements: stakeholder involvement, evidence-informed evaluation, transparency, and appeal mechanisms.^12,13^

 Fourth and lastly: in their key messages, the authors provide several useful suggestions for policy makers, one of which is to provide hospital managers with tools to improve their decision-making. My submission would be that, above all, toolkits need to be practical, with ample attention for the process of decision-making. This would include: involving other relevant stakeholders, with ample room for deliberation; exercising transparency, including making criteria/considerations and underlying value frameworks more explicit for greater responsiveness and accountability; offering techniques to ensure that decisions are informed by evidence; and creating opportunities for health professionals, but probably also patients or the general public, to contest or formally appeal against certain decisions. One suggestion, mentioned in the article’s abstract, deserves some nuance: ‘working with averages’, in an effort to shift the focus of decision-making from the individual patient to a group of patients with a similar condition but simpler (less expensive) treatment requirements. Purely intuitively one could dismiss this suggestion, with the argument that it might provoke (further) ‘protocolisation’ of health care, which many health professionals and patients are wary of, since it would go at the expense of the much needed adaptive expertise in our current health systems and medical practices.^14^

## Conclusion

 In this commentary I hope to have offered some principles, approaches and research methods based on recent work in other areas of health policy research, that may complement the analysis of Waitzberg et al. The issues of conflicting considerations and dilemmas faced by hospital professionals with dual agency will undoubtedly remain pertinent in the foreseeable future in the face of ageing populations, rapid growth of health technologies and fiscal limitations.

## Ethical issues

 Not applicable.

## Competing interests

 Author declares that he has no competing interests.

## Author’s contribution

 LB is the single author of the paper.
